# Influence of non ionizing radiation of base stations on the activity of redox proteins in bovines

**DOI:** 10.1186/1746-6148-10-136

**Published:** 2014-06-19

**Authors:** Michael Hässig, Marietta Wullschleger, Hanspeter Naegeli, Jaqueline Kupper, Bernhard Spiess, Niels Kuster, Myles Capstick, Manuel Murbach

**Affiliations:** 1 Department of Farm Animals, University of Zurich, Winterthurerstrasse 260, CH-8057 Zurich, Switzerland; 2 Institute of Pharmacology and Toxicology, Zurich, Switzerland; 3 Section for Ophthalmology, University of Zurich, Winterthurerstrasse 260, CH-8057 Zurich, Switzerland; 4 IT’IS Foundation ETH Zurich, CH-8092 Zurich, Switzerland

**Keywords:** Bovine, Non ionizing radiation (NIR), Handy antenna, Oxidative stress

## Abstract

**Background:**

The influence of electromagnetic fields on the health of humans and animals is still an intensively discussed and scientifically investigated issue (Prakt Tierarzt 11:15-20, 2003; Umwelt Medizin Gesellschaft 17:326-332, 2004; J Toxicol Environment Health, Part B 12:572–597, 2009). We are surrounded by numerous electromagnetic fields of variable strength, coming from electronic equipment and its power cords, from high-voltage power lines and from antennas for radio, television and mobile communication. Particularly the latter cause’s controversy, as everyone likes to have good mobile reception at anytime and anywhere, whereas nobody wants to have such a basestation antenna in their proximity.

**Results:**

In this experiment, the NIR has resulted in changes in the enzyme activities. Certain enzymes were disabled, others enabled by NIR. Furthermore, individual behavior patterns were observed. While certain cows reacted to NIR, others did not react at all, or even inversely.

**Conclusion:**

The present results coincide with the information from the literature, according to which NIR leads to changes in redox proteins, and that there are individuals who are sensitive to radiation and others that are not. However, the latter could not be distinctly attributed – there are cows that react clearly with one enzyme while they do not react with another enzyme at all, or even the inverse. The study approach of testing ten cows each ten times during three phases has proven to be appropriate. Future studies should however set the post-exposure phase later on.

## Background

The effect of electromagnetic fields associated with mobile telephones on the health of human beings and animals is still controversial (Löscher [1]; for details see SCENIHR [2]) [3,4]. In a former clinical case study [5] a potential association of nuclear cataracts in newborn calves and the installation of a mobile phone antenna on the affected farm was determined. The risk for severe nuclear cataracts in the calves from this farm was 3.5 times higher than the Swiss average. All infectious and toxic etiologies that usually occur in Switzerland were ruled out. Two other publications, based on an investigation on Swiss veal calves, documented relations between the exposure to electromagnetic waves in the frequency range of mobile phone antennas and nuclear cataracts [6] as well as the activity of redox proteins in aqueous humor [7]. The investigation was designed as a field study without standardized conditions. However, the radiated power from an antenna and the distance between antenna and animal are very approximate parameters, since non-ionizing radiation (NIR) propagates very variably dependent on topography and environment and since the animals constantly move towards and away from the antenna. The present study was designed so that the exposure situation was well controlled in order to determine the enzyme activity in a defined setting.

## Results

### GSH-PX

It was found that the phases were significantly different (p = 0.006), however there was no significant difference for the cows (p = 0.534) in the GLM.

**Figure 1:**
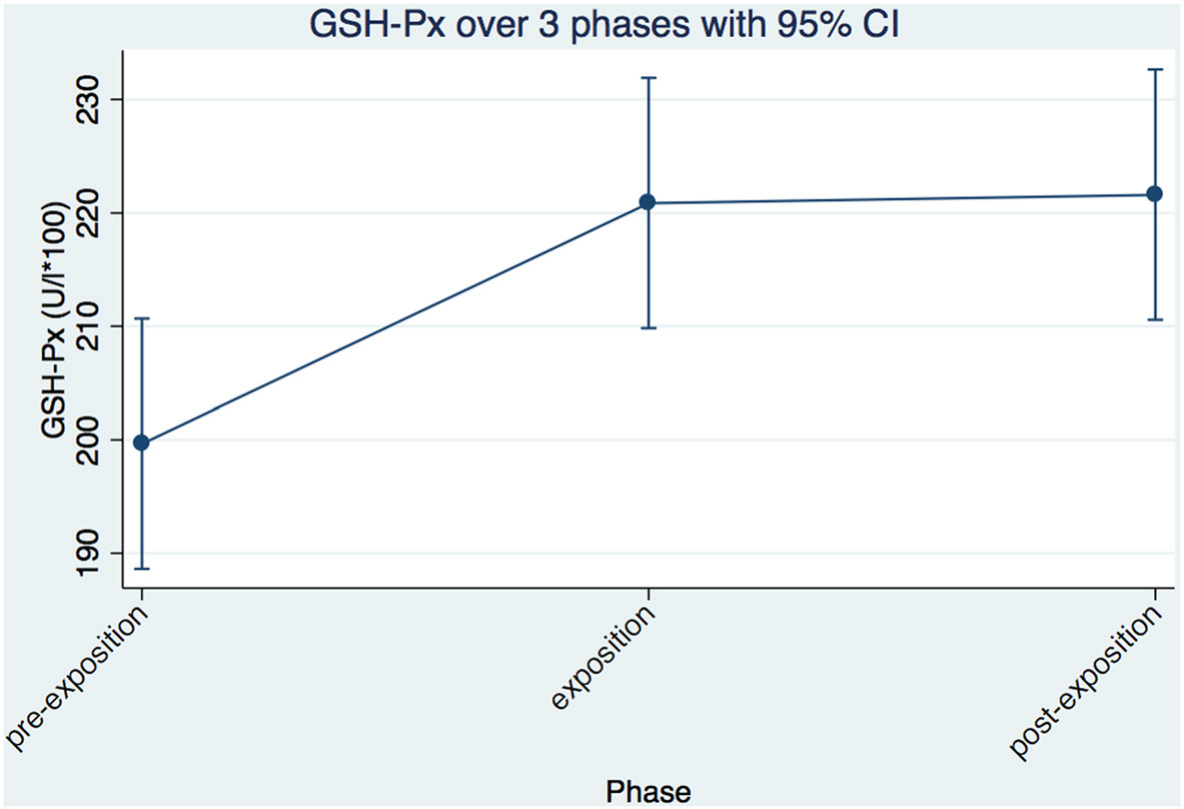
GSH-PX trend of all cows together across the three phases.

Looking at the trend of GSH-PX in the individual cows, it is evident that three cows (Ladina, Karin, Ramosa) showed no effect over time or across the three phases; one cow (Gemsli showed a decrease in activity) between pre-exposure and exposure; and the other six cows (Aline, Palma, Laria, Maya, Regula, Vreni) showed an increase of GSH-PX activity. Between exposure and post exposure, all ten cows remained more or less constant (Table 1). In each phase, each cow was present with 10 individual measurements over 14 days.In summary, the findings of individual cows, show a clear picture, which corresponds to the statistical analyses. The activity of GSH-PX increases between pre-exposure and exposure and remains constant between exposure and post exposure (Figure 1).

**Table 1:** Results the activity of GSH-PX, SOD and catalase before (Pre), at exposure (Exp) and thereafter (Post) to NIS

**Cow**	**Value**	**GSH-PX**			**SOD**			**Catalase**			**Rf-value**		
		**Pre**	**Exp**	**Post**	**Pre**	**Exp**	**Post**	**Pre**	**Exp**	**Post**	**Pre**	**Exp**	**Post**
Aline	Mean	196	228	236	76.7	79.0	67.7	47.2	29.0	25.0	92.0	94.4	103.0
	SEM	18	14	16	1.5	2.1	6.5	8.3	5.4	4.1	9.8	7.2	9.9
	Minimum	125	155	146	69.0	66.2	9.4	12.3	11.7	12.3	46.9	60.8	55
	Median	198	244	263	77.3	80.1	74.3	49.9	26.3	18.9	90.4	97.6	106
	Maximum	289	287	293	87	87.7	76.7	102.0	69.0	46.7	137	129	169
Palma		199	236	236	80.9	82.7	72.1	38.6	34.6	34.5	85.0	99.0	106.0
		18	12	17	1.09	1.2	5.7	8.1	5.9	5.8	7.7	5.4	10.0
		118	176	156	76.3	76.6	32.8	8.22	12.3	16.4	45.4	75.4	58.3
		198	244	262	79.5	83.3	75.8	33.7	29.8	25.1	85.6	96.5	109
		286	283	288	87.8	89.2	96.3	85.7	61.0	71.6	117	131	156
Laria		194	231	235	81	79.6	70.6	41.9	56.2	36.6	85.2	112.0	108.0
		21	16	18	1.31	1.5	6.0	5.7	7.9	3.4	10.6	9.0	9.6
		109	149	135	75.4	74.4	19.8	15.3	31.7	21.4	27.4	66	59.1
		187	240	262	80	77.7	75.1	39.6	50.2	35.8	89.6	121	111
		288	284	294	89	86.3	92.5	79.5	107.0	59.3	124	148	157
Maya		170	214	212	73.4	63.3	68.9	53.5	60.2	40.9	85.7	117.0	101.0
		19	15	20	0.741	9.4	1.0	10.8	9.1	3.6	12.8	8.71	9.7
		105	139	111	69.2	19.5	65.5	17.0	24.6	29.6	41.4	81.2	53.5
		151	219	236	73.7	71.6	67.7	44.5	53.4	36.7	86.7	118	108
		285	275	277	77.2	80	76.5	109.0	106.0	59.9	174	180	136
Regula		186	213	215	80.4	73.1	72.9	50.2	57.9	32.9	86.7	109.0	94.7
		23	14	22	1.1	5.1	1.0	10.5	11.3	3.2	12.0	7.6	10.6
		82.9	133	113	73.2	30	70	6.75	15.3	17.9	34.1	79.6	44
		199	215	252	81.9	75.5	72	42.0	62.5	33.9	87.4	103	111
		290	273	284	84	87.6	80.5	110.0	131.0	48.1	147	151	136
Vreni		169	236	226	75.8	61	70.6	43.5	55.6	28.1	77.8	126.0	98.3
		6	8	14	3.4	9.6	0.8	9.1	8.3	3.2	5.3	9.7	6.8
		137	207	150	53.6	1.5	67.1	12.6	24.6	15.8	60.5	86.4	63.1
		172	233	253	80.2	71.9	70.5	34.5	56.2	29.0	70.7	120	106
		190	269	268	83.6	82.1	74.5	107.0	114.0	45.8	116	186	121
Ladina		208	208	200	72.5	60.6	69.6	42.8	56.6	30.6	97.7	114.0	88.5
		21	16	21	1.1	12.8	2.8	8.1	9.2	3.4	10.1	13.4	10.5
		82	145	99	68	53.3	46.1	13.5	20.2	15.8	29	75.3	36.4
		212	218	217	72.5	72.2	71.1	38.6	49.3	27.1	105	102	97.1
		293	297	270	80.2	80.7	80.5	91.5	105.0	50.2	136	226	128
Karin		236	239	231	81.1	69.6	73.3	57.0	54.8	33.2	114.0	121.0	102.0
		17	16	15	1.4	8.8	1.1	11.7	13.7	3.2	5.4	10.3	7.2
		122	125	154	74.9	8.39	68.5	17.0	4.4	22.0	83.3	75.7	67.3
		266	255	256	81.2	75.6	72.9	50.2	44.7	30.5	118	124	113
		293	288	267	89.0	87.5	81.3	130.0	156.0	57.8	133	174	124
Ramosa		216	213	218	79.7	74.3	78.1	53.0	68.7	36.3	103.0	114.0	94.6
		20	18	21	1.2	5.6	2.6	7.4	13.7	2.7	11.2	11.4	9.4
		93	130	112	75.4	26.1	71.7	19.7	19.4	26.7	30.7	63.2	53.9
		237	206	244	78.5	78.2	74.9	51.5	66.5	34.6	105	109	106
		293	290	284	88.8	88.1	96	90.7	169.0	50.5	165	195	125
Gemsli		222	190	206	73.4	79.1	78.4	61.9	54.3	47.0	115.0	92.1	95.5
		23	20	21	5.4	2.3	2.1	8.8	5.1	4.7	14.7	10.0	9.3
		90.7	114	103	25.3	63	72.2	26.7	27.6	20.2	32.3	50.9	46.8
		248	176	231	78.6	80	76.4	66.5	54.3	47.5	124	80.3	109
		298	289	280	82.5	88.4	94.7	104.0	77.2	73.1	173	140	130

Each cow was tested ten times within each time period. The individual cells show in a row mean, standard error of mean (SEM), minimum, median, maximum. For the calculation of the Rf-value refer to material and methods.

### SOD

**Figure 2:**
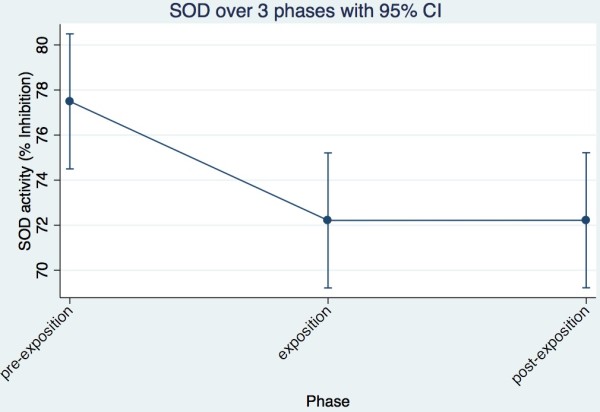
SOD trend of all cows together across the three phases.

The data proved not to be normally distributed and there was no suitable transformation. The GLM showed that both the phases (p = 0.019) and cows (p = 0.025) were significantly different. It was obvious that six cows (Ladina, Karin, Ramosa, Maya, Regula, Vreni) showed a decrease in SOD activity between pre-exposure and exposure. Between exposure and post exposure, 5 cows were increasing the activity of the SOD again (Ladina, Karin, Ramosa, Maya, Vreni) and one cow remained constant (Regula). Of the remaining four cows, three (Aline, Palma, Gemsli), showed an increase of activity between pre-exposure and exposure and in 3 cows activity decreased between exposure and post-exposure (Aline, Palma, Laria). In one cow the activity remained constant between pre-exposure and exposure (Laria) and in another (Gemsli) between exposure and post-exposure (Table 1).Summarizing the findings of individual cows, an image corresponding to the statistical analysis arises. The activity of SOD decreases between pre-exposure and exposure and remains constant between exposure and post-exposure (Figure 2).

### Catalase

**Figure 3:**
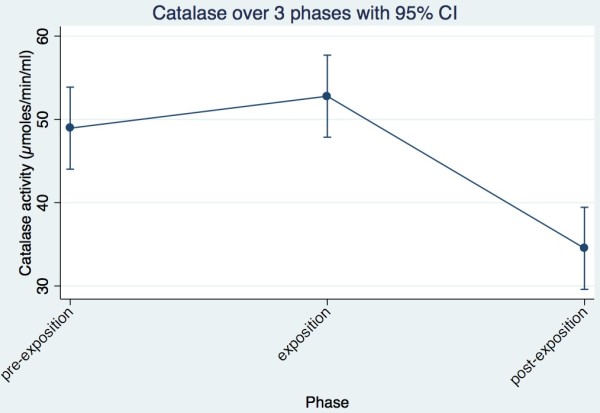
Catalase trend of all cows together across the three phases.

The logarithmic transformation of the catalase values proved to be approximately normally distributed. The GLM showed that the phases (p = 0.050) were significantly different. The cows, however, showed no significant difference (p = 0.158). Looking at the trend of catalase in the individual cows, it is evident that six cows (Ladina, Laria, Ramosa, Maya, Regula, Vreni) showed an increase in activity between pre-exposure and exposure. These six cows then showed a reduction of activity between exposure and post-exposure. However, four cows (Aline, Palma, Gemsli, Karin) showed a decrease in activity between pre-exposure and exposure. These four cows showed a further decline of activity (Aline, Gemsli, Karin) or remained constant (Palm) between exposure and post exposure (Table 1).In summary, the catalase activity of individual cows makes a clear picture, which corresponds to the statistical analysis. The activity of catalase increases slightly between pre-exposure and exposure (not significantly) decreases and significantly between exposure and post-exposure (Figure 3).

### Individual response

Table 2 shows the controversial situation that there are NIR-sensitive, as well as NIR-non-sensitive cows. Cows representing the majority of a certain time course over the three phases were referred to as reagent. Cows representing an alternative time course over the three phases were referred as inverse reagents. Those without response to NIR were referred to as non-reagents. Although there are cows, which reacted with all the three enzymes (Maya, Regula, Vreni), as well as one cow, which responded inverse with all three enzymes (Gemsli), the other six cows showed no consistent pattern of response.

**Table 2:** Reagents, non-reagents and inverse reagents for GSH-PX, SOD and catalase after exposure to NIS (for categorization see text)

**Cow**	**GSH-PX**			**SOD**			**Catalase**		
	**Reagent**	**Non-reagent**	**Inverse reagent**	**Reagent**	**Non-reagent**	**Inverse reagent**	**Reagent**	**Non-reagent**	**Inverse reagent**
Aline	X					X			X
Palma	X					X		X	
Laria	X				X		X		
Maya	X			X			X		
Regula	X			X			X		
Vreni	X			X			X		
Ladina		X		X			X		
Karin		X		X				X	
Ramosa		X		X			X		
Gemsli			X			X			X

### Summary evaluation

**Figure 4:**
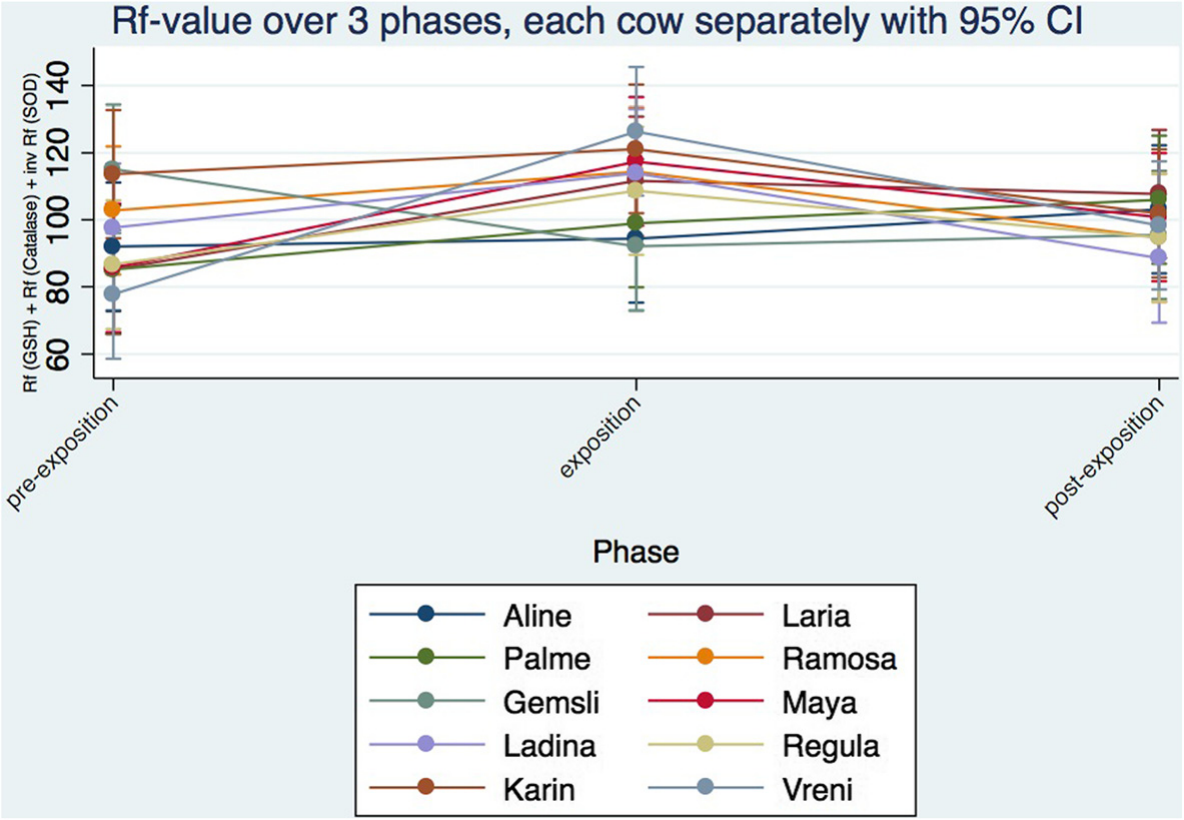
**Trend of the summarized Rf-value for GSH-PX, Catalase and the inverse SOD value for each individual cow across the three phases.** For calculation of the Rf-value refer to materials and methods.

**Figure 5:**
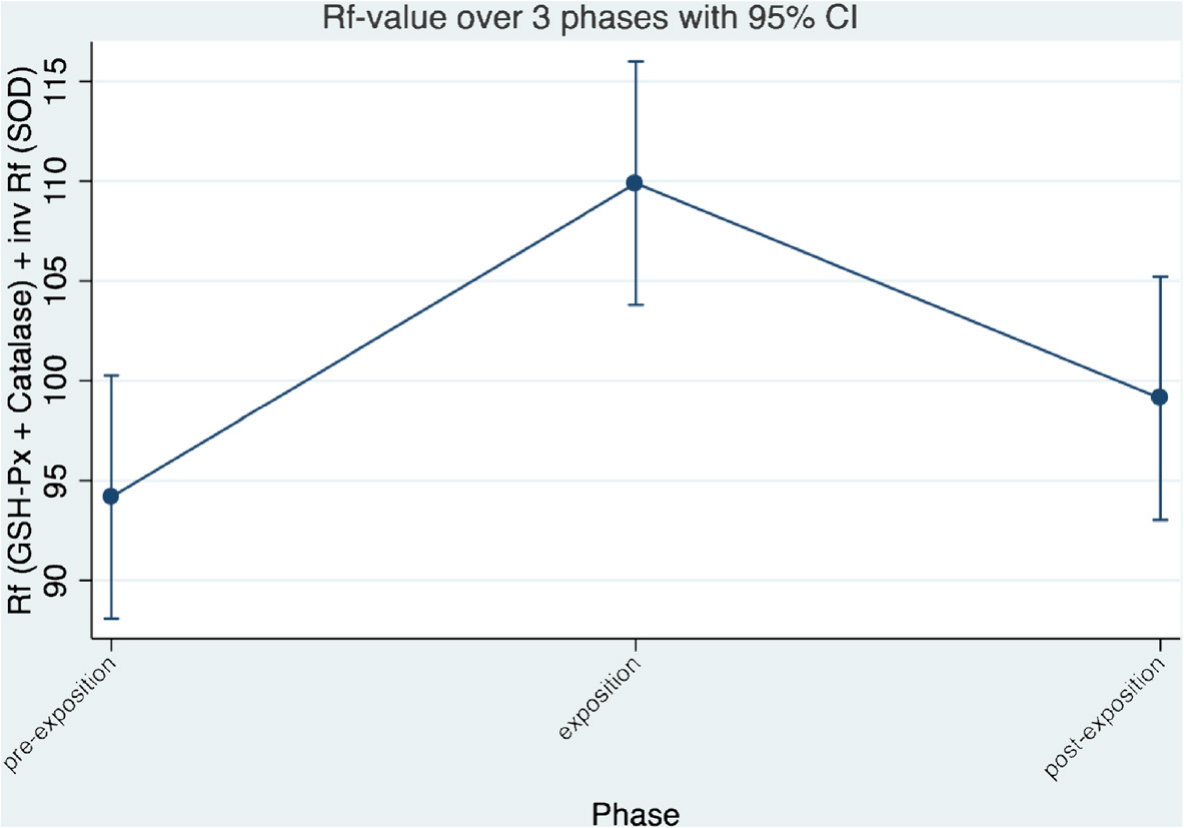
**Trend of the summarized Rf-value for GSH-PX, Catalase and the inverse SOD value of all cows together across the three phases.** For calculation of the Rf-value refer to materials and methods.

To compare the individual enzyme values directly, a Rf-transformation or standardization was made with the range as 100 units for each parameter. As SOD runs like a mirror image to the horizontal compared to GSH-PX and catalase, the inverse (complementary value to 1) of the Rf-value has been used in the overall model for SOD. The Rf-data is normally distributed. The GLM showed that the phases (p < 0.001) differ significantly, but not the cows (p = 0.628). The Rf value of GSH-PX, catalase and inverse SOD differs between the pre-exposure and the exposure phase (p = 0.001) and between the exposure and the post-exposure phase (p = 0.044) in a factorial analysis of variance (p = 0.0014) with Bonferroni test. No difference however between the pre-exposure and the post-exposure period (p = 0.785) was found. The trend of the Rf-value of GSH-PX, catalase and inverse SOD in the individual cows shows that seven cows (Ladina, Karin, Laria, Ramosa, Maya, Regula, Vreni) between pre-exposure and exposure show an increase of Rf-values and a decrease of the same between exposure and post-exposure. Three cows (Aline, Palma, Gemsli) showed no or an inverse effect (Figure 4) across the three phases. In this presentation each cow contributes 30 values for each phase (10 each for SOD, GSH-PX and catalase) in accordance with the study protocol, which has massively increased the power of the last statistics.Summarizing the findings of individual cows, a clear image corresponding to the statistical analysis is given. The combined Rf-values of catalase, GSH and the inverse SOD increased between pre-exposure and exposure and declined between exposure and post-exposure but not between cows (Figure 5).

## Discussion

In this study, NIR has resulted in significant changes in the activities of the investigated redox enzymes. The activity of GSH-PX increased significantly between pre-exposure and exposure period, whereas the SOD recorded a significant decrease in the same trend in activity and is in accordance with Eltiti [8]. The Catalase activity increased only slightly between pre-exposure and exposure period. Between exposure and post exposure period, the Catalase activity showed a significant decrease, while the activities of GSH-PX and SOD remained largely constant. The individual cows showed only for SOD a significant difference in activity. The phases had significant impact on the results in all three enzymes.

In SOD, GSH-PX and catalase a temporal trend is unlikely. If a temporal trend would be postulated, a continuous increase or decrease would have been observed over the three phases. It can be concluded with great probability that the exposure to non-ionizing radiation (NIR) has led to a significant increase of GSH-PX activity and is in accordance with earlier findings by Hässig et al. [5,7]. A seasonal time trend can be ruled out by the short alterations in the summary evaluation. The SOD showed a significant reduction in activity. However, whether this change of enzyme activity is reversible or irreversible could not be determined conclusively on the basis of this data. The enzyme activities remained more or less unchanged during the post exposure period. To confirm this, the measurements of post-exposure must take place later.

No significant change in the Catalase activity could be determined between pre-exposure and exposure. However, a significant decrease of the Catalase activity is visible between exposure and post-exposure. A possible explanation for the different behavior of the measured enzymes is their respective up and down regulation of the pH, according to the pK_a_ formula (-log_10_*K*_a,_ where K_a_ is the acid dissociation constant).

A temporal trend can definitely be excluded based on the aggregated Rf-values of Catalase, GSH-PX and the inverse Rf-values of SOD. It can be said with great probability that the exposure to non-ionizing radiation (NIR) has led to a significant change in the overall activity of the three measured redox enzymes.

Confounders may be largely excluded. The cows were in a barn with low background radiation. The NIR exposure has been standardized under controlled conditions. Because the experiment was conducted in the winter, the animals spent most of the time in their cubicles in the barn and were fed uniformly. They were cared for by the usual staff and at the usual times. Ambient conditions such as stable temperature and humidity were largely constant. The cows were healthy during the entire trial period, apart from two cases of mastitis. Mastitis can lead to alterations in redox enzymes due to the higher production of free radicals [9]. Collecting blood from the tail vein is also a low-stress method: most of the cows remained undisturbed during blood collection.

The test sequence and the study approach, ten cows during three phases to be sampled, has proved useful. Only the post-exposure period should be extended in future trials, to allow more specific statements regarding reversibility. Even if a significant difference between the exposure and the post-exposure phase was found in the comprehensive evaluation of all three enzymes, it must be remembered that catalase in the individual evaluation was the only enzyme, of which the activity has dropped significantly between the exposure and the post-exposure phase.

It is often heard in connection with the possible influence of NIR on the health of humans and animals, that there are radiation-sensitive, as well as non-radiation-sensitive individuals. In humans, this phenomenon was studied already [8]. This controversial assertion could not be demonstrated in this experiment. Although there were trends in cows, which reacted with all three appropriate enzymes (Maya, Regula, Vreni), as well as one cow, which responded inversely with all three enzymes (Gremsli). A correlation between breed, age, reproductive status, and diseases with NIR sensitivity was not found (Tables 2 and 3). The other six cows showed no consistent pattern of response. Regarding NIR sensitivity, a reliable statement cannot be made. More animals should be investigated to find a definite answer to this question. This could be the subject of a future study.

**Table 3:** Information about the individual cows

**Cow**	**Age (years)**	**Breed**	DIM*	**Reproductive status on Nov. 30**^**th**^**2012**	**EBL**	**IBR**	**BVD**	**SBV**	**Specials**
Aline	12	Brown Swiss	225	Empty	neg	neg	pos	pos	none
Palma	7	Simmental	274	Pregnant	neg	neg	pos	pos	none
Laria	4	Simmental	282	Pregnant	neg	neg	neg	pos	none
Maya	9	Brown Swiss	180	Pregnant	neg	neg	pos	pos	none
Regula	9	Brown Swiss	220	Empty	neg	neg	pos	pos	Mastitis*** (A, B, C, D, C. bovis) Nov. 13^th^ 2012, Abortion
Vreni	9	Brown Swiss	80	Pregnant	neg	neg	pos	pos	none
Ladina	3	Brown Swiss	264	Pregnant	neg	neg	pos	pos	none
Karin	4	Simmental	134	Empty	neg	neg	pos	pos	AI** Nov. 20^th^ 2012, Jan. 3^rd^ 2013
Ramosa	13	Brown Swiss	256	Pregnant	neg	neg	pos	pos	Mastitis*** (A, C, S. uberis) Dec. 29^th^ 2012
Gemsli	6	Brown Swiss	304	Empty	neg	neg	neg	pos	none

*DIM: Days in milk at beginning of first period, November 30^th^ 2012 (1.2.13).

**AI: Artificial insemination.

***: Infected quarter, bacteria.

EBL: Enzootic bovine leucosis.

IBR: Infectious bovine rhinotracheitis.

BVD: Bovine virus diarrhea (antibody-test, all cows free of virus).

SBV: Schmallenberg Virus (antibody-test, all cows free of virus).

Humans and animals interact constantly with the living and non-living environment, during which the individual tries to maintain its homeostasis. This happens for example when heat exposure through cooling mechanisms is controlled for temperature homeostasis. For NIR exposure it is similar: the cow tries to counteract NIS exposure, which affects their redox status, by regulating enzyme activity, to ensure homeostasis of the pH in the body. If the individual is able to hold the values to regulate within the physiological limits, the individual is not ill. If the regulatory functions of the body are overwhelmed, the individual gets ill. In our case, a range for GSH-PX of 82.29 to 297.59 U/l was measured. The changes of GSH-PX in our study are within the physiological range, as specified in the literature (Smith [10]; 25 to 500 U/l). Useful values for comparison of SOD and catalase were not found. Thus, influences of NIR are detectable in the presented study but no outliner could be determined for GSH-PX in non-physiological areas at any time. Further studies on bovines have to be blinded and even more standardized by means of breed and gestation synchronization. For such a study much more money must be available than for the presented one.

## Conclusion

NIR has resulted in changes in the enzyme activity in the presented study. Certain enzymes are disabled others enabled by NIR. The activity of GSH-PX increased whereas the one of SOD decreased. The present results coincide with the information from the literature that NIR will lead to changes in redox proteins and that there are radiation-sensitive, as well as non-radiation-sensitive individuals.

The study approach with 10 blood samples per cow and phase (pre-exposure, exposure and post-exposure) has proved appropriate.

To make a reliable statement regarding NIR reactivity, more animals should be tested.

In future studies, the post exposure period should be extended, the exposure has to be blinded and even more standardized by means of breed and gestation synchronization.

## Methods

### Animals and experimental setting

**Figure 6:**
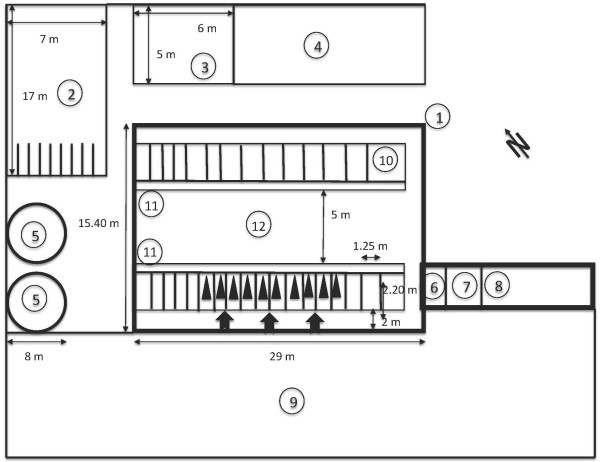
**Map of the study location.** 1: Main cowshed made of concrete and wood. 2: Horses. 3: Meet calves. 4: Sheep. 5: Silo. 6: Passage. 7: Dairy. 8: Toolshed. 9: Yard – area where cows are turned out. 10: Goats. 11: Feeding table. 12: Drive thru ,  Tab: Antenna.: Cow in trial.

A controlled case–control study where the cases served sequentially as their own control was performed. Ten cows of different ages standing next to each other have been randomly selected and chosen at different stages of gestation (Table 3). The study was conducted under the research animal approval of the Veterinary Office of the canton of Zurich (No. tv 4975). Furthermore, the in vivo experiment followed in all aspects the guidelines checklist of ARRIVE (http://www.nc3rs.org.uk/downloaddoc.asp?id=2093&page=1357&skin=0, June 4^th^, 2014) (Additional file 1). The experiment was conducted at the field station “Stigenhof” of the animal hospital in Oberembrach near Zurich (coordinates: 47.4974065,8.6530844). The cows were in tie stalls so that their exact position could be controlled. An overview/scheme of the cowshed with the cows and the antennas in place is provided in Figure 6. The experiment was conducted between the end of November 2012 until early February 2013, because the animals spend most of the day indoors at this time of year (no grazing; 2 hours turnout per day on a paved square in front of the barn in accordance with requirements of the Federal Animal Welfare Act) and because the winter feeding is constant. Furthermore, the test location “Stigenhof” is known for poor cell phone coverage especially in the cowshed. Therefore a very low background radiation could be assumed and was later on assessed.

The cows were cared for by the usual staff. They were fed and milked twice daily at the same times. The feeding was constant. It consisted of hay and a total mixed ratio (TMR) of grass and corn silage, ensiled sugar beet chips and mineral salt. The cows were turned out on a paved square in front of the barn 2 h per day in accordance with the Swiss Animal Protection law.

During the trial, two cows had to be treated for mastitis, 3 cows were dried off and one gave birth. Other than that the cows were healthy and showed no behavioral abnormalities.

### Exposure set-up

**Figure 7:**
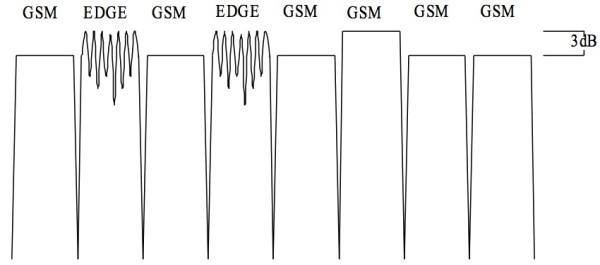
A GSM-Frame (4.62 ms) of the used mobile phone basis station signal.

**Figure 8:**
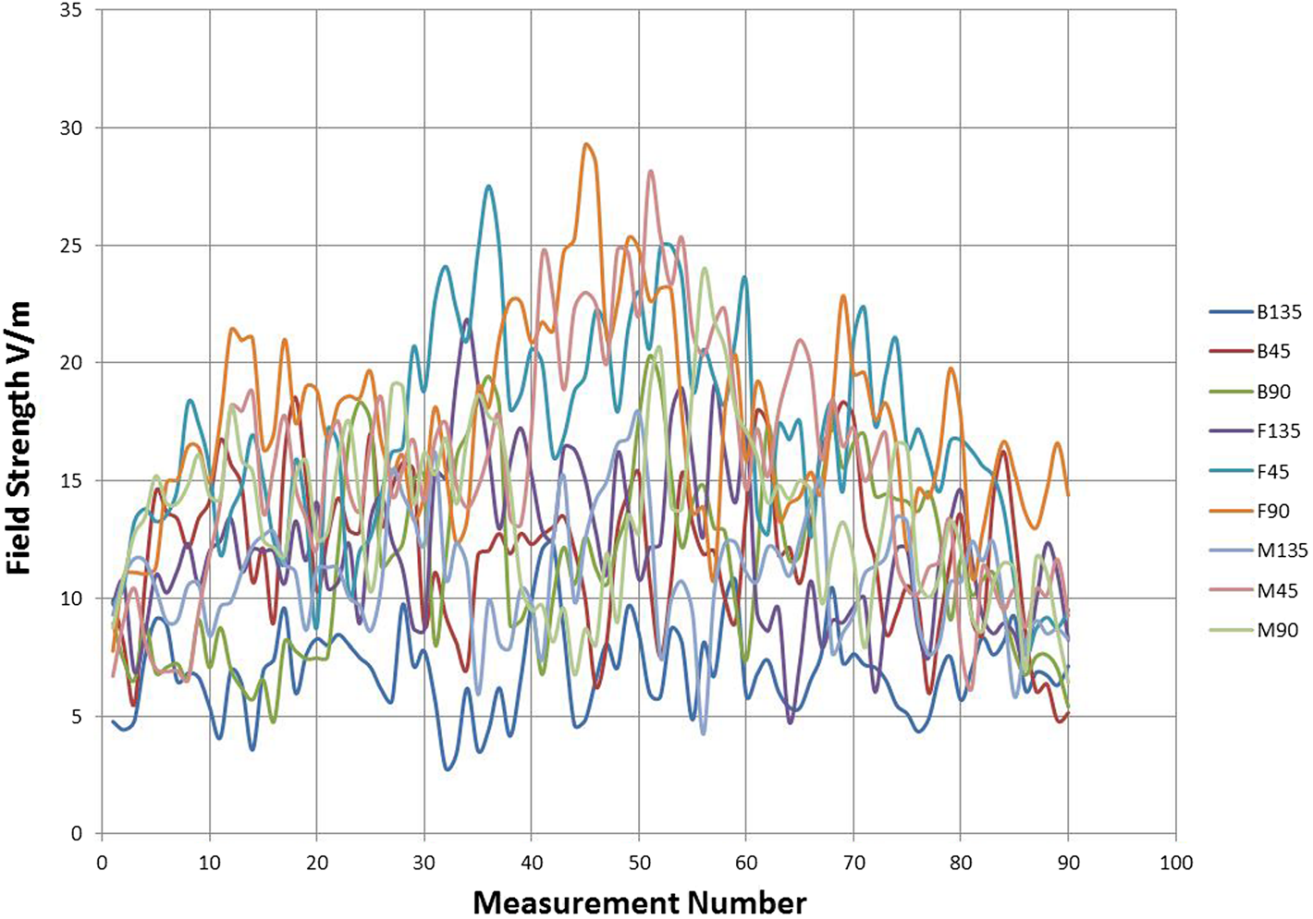
The average field strength and standard deviation of the incident field at each cow location.

**Figure 9:**
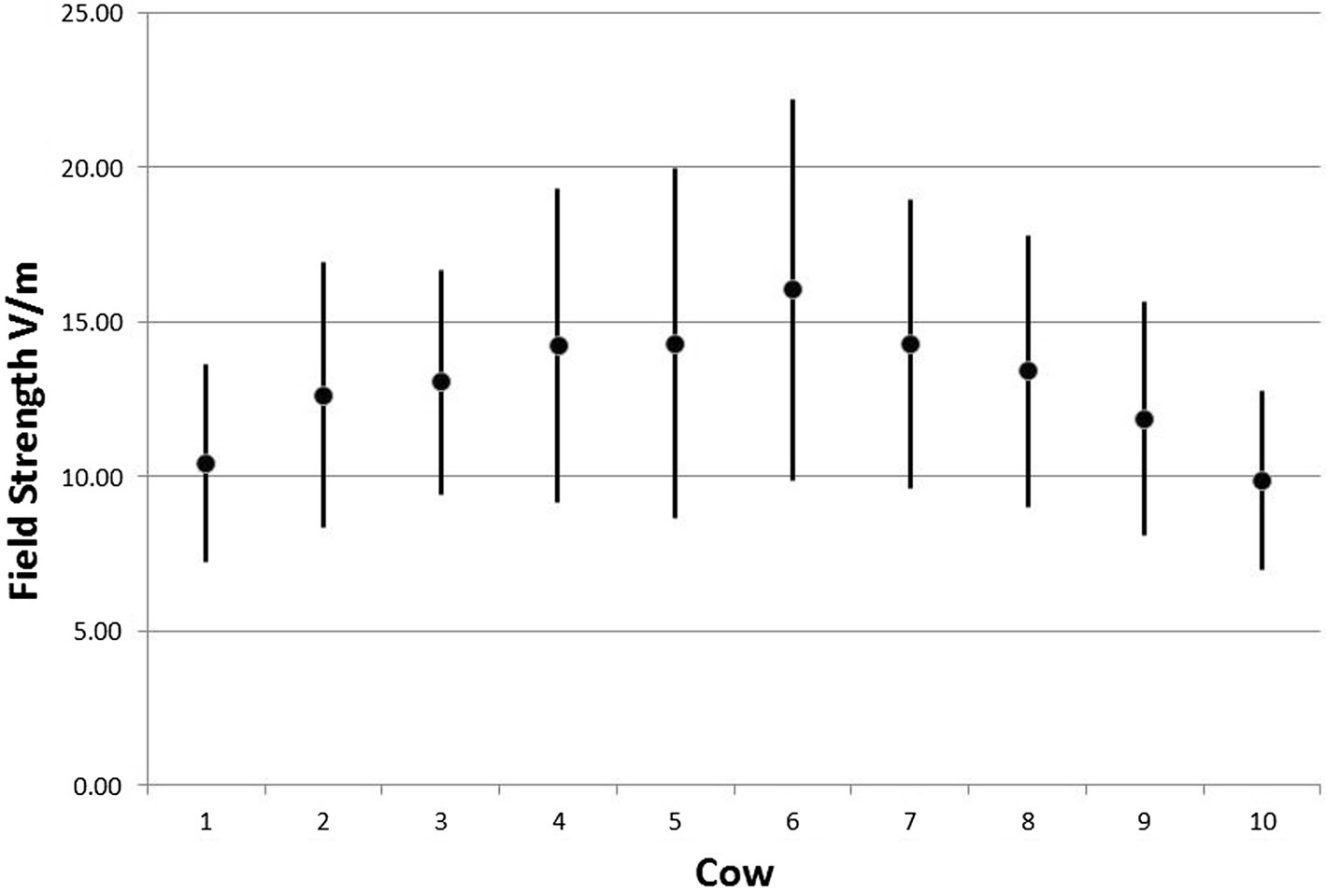
Total field (vector sum of X, Y and Z components).

The non-ionizing radiation (NIR) exposure equipment was provided by IT'IS Foundation of ETH Zurich (Eidgenössische Technische Hochschule; Federal Institute of Technology). The exposure was from three 900 MHz-antennas (SPA 920/65/9/0/V, Huber & Suhner, Herisau, Switzerland) installed at 2 m height behind the ten cows with a 30° downward tilt and not reachable either by the cows or staff. The signal was similar to a GSM base station, with 5 GSM (voice) and 3 EDGE - (data) pulses within each of the 4.62 ms repetitive GSM frames (Figure 7). One of the GSM-pulses used was 3 dB higher, to simulate the control pulse. While the EDGE pulses show amplitude variation, due to the 8 PSK modulation used. In total, a peak-to-average ratio of 2.2 (3.4 dB) resulted. The Federal Office of Communication (BACOM) allocated an experimental license defining the carrier frequency of 916.5 MHz and the maximum effective radiated power, which in turn defined the maximum field strength available at the cow locations. The field strength was measured in the empty stalls where the ten cows would be located. It should be mentioned that the field distribution will change when the cows are present in the stalls. Reflections that were present in the empty environment may be damped, while additional reflections from the animals are introduced changing the standing wave pattern in sympathy with the cow movement. The incident field for all ten cows was on average 12 V/m, with a standard deviation of 35%. The average field strength and standard deviation of the incident field at each cow location is based upon ~80 measurements. The measurements were performed on 9 tracks, the E-field probe was scanned along each track collecting data as it was scanned. The tracks were at three locations, at the front (F) middle (M) and back (B) of the stall and at three heights, namely 45 cm, 90 cm and 135 cm, giving the total of 9 different tracks. The data for the total field in V/m (vector sum of X, Y and Z components) can be seen in Figure 8. The data clearly shows the multipath/reflection impact on the field homogeneity. Figure 9 shows the average and SD (shown as the error bars) of the incident field for each cow. The overall average 12 V/m and SD 35% are based on all 810 individual measurements, while the highest individual measured value was 29 V/m and lowest 3.4 V/m. The average field strength was therefore below international limit set by ICNIRP (41 V/m), but well above the Swiss limit for and individual base station of 4.1 V/m (ONIR). NIR exposure took place only, when no staff were in the stable. Warning lights were installed at the two stable doors as well as a switch at the main entrance where the exposure could be turned off. The data for the antenna transmit power as well as temperature and humidity were recorded by a data logger during the entire trial. The exposure times during the 30 days irradiation period resulted in a daily exposure between 6 h 36 min and 19 h 00 min (on average 16 h 43 min, standard deviation 3 h 03 min).

The study was not blinded at this stage due to the short allowance for exposure by the BACOM and due to reach stable conditions during wintertime.

### Procedure

**Figure 10:**
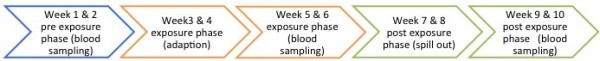
Trial.

The trial lasted for a total of 10 weeks (Figure 10). Between 10 to 14 days prior to the exposure to radiation, ten blood samples, one per day from the tail vein (Vena coccygea) of each cow were taken to obtain control values of enzyme activities (pre exposure phase). After this the transmitter was active for 4 weeks. The first 14 days were selected as the adaptation phase followed by a 14-day test phase (exposure), during which again 10 blood samples were taken from each cow. Then, the system was turned off. The cows had a 14-day break before one last time within 14 days 10 blood samples were taken (post exposure period).

The antenna ran from Tuesday, 11 December 2012, 14:30 to Wednesday, 9 January 2013 at 14:30 without any problems. Only on Saturday, December 15th the 3G router had to be rebooted because the Internet connection to the control computer was interrupted.

On each sampling day 16 ml blood were taken per cow:

•10 ml without additives for the production of serum for the measurement of the SOD (superoxide dehydrogenase)

•2 ml of fluoride for the production of erythrocyte concentrate for the measurement of GSH-PX (glutathione peroxidase) and CAT (catalase)

•4 ml EDTA (Vacuette®, ref. 454222, Greiner Bio-One, Austria) for reserve purposes.

The blood samples were randomly labeled and stored until processing at 4°C (randomization and blinding). The serum and erythrocyte concentrates have been frozen until the measurements at -18°C.

The measurement of GSH-PX was performed by the “Glutathione peroxidase cellular activity assay Kit” (Sigma Aldrich) on a “Cobas Mira S” (Roche).

The measurement of the SOD was performed using the “SOD determination Kit” (Sigma Aldrich) and the microplate reader “MRX II” (Dynex technologies).

The measurement of catalase was performed with the “catalase assay Kit” (Sigma Aldrich) and the spectrophotometer “Ultrospec 2100 pro” from Amersham Biosciences).

### Statistics

The statistical analyses were performed using the program STATA (StataCorp., 2011; Stata statistical software: Release 12.1; College Station, TX, United States: StataCorp LP). The data has been checked for normality<swilk>using the Shapiro-Wilk test. These < ladder > - or<gladder>tests were used for the best possible transformation for not normally distributed data if possible. The descriptive analysis encompassed median, confidence interval 95% (CI 95%), minimum, maximum, standard deviation and standard error, depending on the respective distribution. The figures show the mean and the 95% CI. Furthermore, generalized linear, hierarchical regression models (GLM) were calculated to meet the repeated measurements of the cows in the three study periods < xtmixed parameter cow phase age breed DIM time-point || time-point: || cow:>. Time-point and cow where the repeated independend variables in the model. Age, breed DIM (days in milk) and time-point were skipped in the presented model, because they did not substantially influence the results (p > 0.05). To compare the individual blood values directly, an Rf (relative frequency) transformation or standardization was undertaken whereby the range was set as 100 units for each parameter. As SOD runs mirrored to a horizontal line compared to GSH-PX and catalase, the inverse value of the Rf has been chosen in the overall model for SOD. A p-value of ≤ 0.05 was accepted as significant. A p-value between 0.05 and 0.2 was accepted as a trend.

## Competing interests

The authors declare that they have no competing interests.

## Authors’ contributions

MH initiated and planned the study and prepared the manuscript. MW carried out the data collection and analysis for her master thesis under supervision of MH. HPN selected the analyzed enzymes and organized the laboratory analysis. JK helped and supervised MW with the laboratory analysis. BS has proof read and planned the study. MM established the setup antenna, MC calculated and measured the radiation of the antenna. NK set up the antenna experiment. All authors read and approved the final manuscript.

## Supplementary Material

Additional file 1 The ARRIVE guidlines checklist. Click here for file

## References

[B1] Löscher W Die Auswirkungen elektromagnetischer Felder von Mobilfunksendeanlagen auf Leistung, Gesundheit und Verhalten landwirtschaftlicher Nutztiere: Eine Bestandsaufnahme Prakt Tierarzt 2003 11 15 20

[B2] SCENIHR (Scientific Committee On Emerging and Newly Identified Health Risks) Possible effects of Electromagnetic Fields (EMF) on Human Health 2007 http://ec.europa.eu/health/ph_risk/committees/04_scenihr/docs/scenihr_o_007.pdf 10.1016/j.tox.2008.02.004 18453044

[B3] Eger H Hagen KU Lucas B Vogel P Voit H Einfluss der räumlichen Nähe von Mobilfunksendeanlagen auf die Krebsinzidenz Umwelt Medizin Gesellschaft 2004 17 326 332

[B4] Van Rongen E Croft R Juutilainen J Lagroye I Miyakoshi J Saunders R De Seze R Tenforde T Verschaeve L Veyret B Effects of radiofrequency electromagnetic fields on the human nervous system J Toxicol Environment Health B Crit Rev 2009 12 572 597 10.1080/10937400903458940 20183535

[B5] Hässig M Jud F Spiess B Vermehrtes Auftreten von nukleärem Katarakt nach Erstellung einer Mobilfunkbasisstation Schweiz Arch Tierheilkd 2012 154 82 86 22287140 10.1024/0036-7281/a000300

[B6] Hässig M Jud F Nägeli H Kupper J Spiess B Prevalence of nuclear cataract in Swiss veal calves and its possible association with mobile telephone antenna base stations Schweiz Arch Tierheilkd 2009 151 471 478 19780007 10.1024/0036-7281.151.10.471

[B7] Hässig M Jud F Nägeli H Kupper J Spiess B Einfluss von Mobiltelefon-Basisstationen auf die Aktivität der GSH, SOD und Catalase im Augenkammerwasser von Kälbern Klauentierpraxis 2012 20 133 137

[B8] Eltiti S Wallace D Ridgewell A Zougkou K Russo R Sepulveda F Mirshekar-Syahkal D Rasor P Deeble R Fox E Does short-term exposure to mobile phone base station signals increase symptoms in individuals who report sensitivity to electromagnetic fields? A double-blind randomized provocation study Environ Health Perspect 2007 115 1603 1608 18007992 10.1289/ehp.10286 PMC2072835

[B9] Spector A Oxidative stress-induced cataract: mechanism of action FASEB J 1995 9 1173 1182 7672510

[B10] Smith B Large Animal Internal Medicine 1996 2 Missouri, USA: Mosby-Year Book Inc

